# Needless Pets

**Published:** 1916-08

**Authors:** 


### Needless Pets.

Dogs, cats, rats and mice are the mediums through which much disease is scattered. People are constantly making war on rats and mice from the economic necessity, but they seldom think of them as disease carriers and germ spreaders. There is no possible good that can come from rats and mice. It is more expensive to maintain a few of them about a place than to feed another person, aside from the harm they do in spreading sickness. They should be exterminated even by national, State and municipal action, if that be necessary. New Orleans and some other cities are endeavoring to do this for two reasons: that they are expensive to maintain and that they are responsible for some of the public scourges. The time will come when a city will be ashamed to admit that it has rats or mice in it.

But what about the dogs and cats? They are even worse than the rats and mice, for they are the favorite household pets of the country. They not only have free access to many homes, but are taken into the arms of the women and children of the family, and fondled and caressed, often when they are loaded with disease germs. Dogs and cats are innately filthy. Nothing is too dirty for them to get into with their feet and mouths; no place terrifies them because of the diseases that may be lurking there. They delight to roam about questionable places, especially at night, and expect to receive their customary caresses the next day. They are expensive to feed and dangerous to have around. Why will sensible people keep them? This may be regarded as a subject foreign to the scope of a medical journal, but it is written in the hope that the physicians of the country may interest themselves in exterminating these useless household pests.

